# Comparative Transcriptome Analyses Reveal the Mechanisms Underlying Waterlogging Tolerance in Barley

**DOI:** 10.3390/plants14010028

**Published:** 2024-12-25

**Authors:** Juan Zhu, Haoxin Yin, Cong Cao, Chengqun Sun, Mengna Zhang, Yi Hong, Yuhang Zhang, Chao Lv, Baojian Guo, Feifei Wang, Rugen Xu

**Affiliations:** Key Laboratory of Plant Functional Genomics of the Ministry of Education, Jiangsu Key Laboratory of Crop Genomics and Molecular Breeding, Jiangsu Co-Innovation Center for Modern Production Technology of Grain Crops, Joint International Research Laboratory of Agriculture and Agri-Product Safetyof Ministry of Education of China, Yangzhou University, Yangzhou 225009, China; 007670@yzu.edu.cn (J.Z.); 15332889179@163.com (H.Y.); 18151151192@163.com (C.C.); jujin777888@outlook.com (C.S.); zmnkarry@163.com (M.Z.); 008733@yzu.edu.cn (Y.H.); 17625434925@163.com (Y.Z.); clv@yzu.edu.cn (C.L.); bjguo@yzu.edu.cn (B.G.); feifei.wang@yzu.edu.cn (F.W.)

**Keywords:** waterlogging tolerance, transcriptome analysis, differentially expressed genes, KEGG analysis, candidate genes

## Abstract

Waterlogging is becoming a global issue, affecting crop growth and yield in low-lying rainfed areas. A DH line, TamF169, showing superior waterlogging tolerance, and its waterlogging-sensitive parent, Franklin, were used to conduct transcriptome analyses. The results showed that 2209 and 2578 differentially expressed genes (DEGs) in Franklin and 1997 and 1709 DEGs in TamF169 were detected by comparing gene expression levels under control and waterlogging after 4 and 8 days, respectively, with 392 and 257 DEGs being specific to TamF169 after 4 and 8 days under waterlogging, respectively. KEGG analysis showed that glycolysis/gluconeogenesis, the MAPK signaling pathway, plant hormone signaling, and galactose metabolism pathways were significantly enriched in the waterlogging-tolerant genotype TamF169 four days after waterlogging. The qPCR results were consistent with the transcriptome data, suggesting the reliability of the transcriptome sequencing. A total of 13 genes in the mapping region of a QTL for root cortical aerenchyma (RCA) showed different expression levels in Franklin or TamF169, and the potential candidate genes for *RCA−QTL* are discussed. This study offers valuable information on the mechanism of tolerance to waterlogging stress in the DH line TamF169 and provides the candidate genes for *RCA−QTL*.

## 1. Introduction

In recent decades, the unpredictability of climate change and the frequent occurrence of extreme weather events have led to a global increase in waterlogging incidents in agricultural fields [1,2]. It is estimated that 10 to 16% of arable soil worldwide is affected by waterlogging (FAO, 2015, http://www.fao.org/3/a-i5128e, accessed on 2 December 2024). The yield penalties caused by waterlogging will increase from 3–11% historically to 10–20% by 2080 [3]. The most cost-effective way to address the effects of waterlogging damage is to improve the plant’s resistance to waterlogging [4]. Therefore, it is important to study crop waterlogging tolerance and its underlying mechanisms to support breeding efforts for waterlogging-tolerant crops.

The root system is the first target of plant waterlogging stress. Under waterlogged conditions, the exchange of O_2_ and CO_2_ in the root system is inhibited, thus creating hypoxic or even anoxic conditions, thus impeding respiration. As a result, roots are unable to grow properly, or even die, which negatively affects its growth and the development of eventual yields [5]. Hypoxic or even anoxic conditions, including the impairment of water and nutrient absorption, and the balance of hormone and reactive oxygen species (ROS), cause physiological dysfunction, resulting in stunted growth. Low oxygen changes the soil electrochemical composition, resulting in higher concentrations of toxic elements, including manganese (Mn^2+^), iron (Fe^2+^), and sulfide (H_2_S, HS^−^, S^2−^) [6]. The outward diffusion of ethylene (C_2_H_4_) in plants is impeded under waterlogged soil, and the elevated ethylene level is potentially a dangerous molecule that inhibits root elongation and growth [7].

Barley (*Hordeum vulgare* L.) is the fourth cereal crop worldwide and relatively more sensitive to waterlogging stress compared with other crop species [8]. The identification of quantitative trait loci (QTL) associated with waterlogging tolerance in barley opens up possible avenues for marker-assisted selection and the discovery of potential genes for further work. Xu et al. [9] summarized the QTL associated with waterlogging tolerance identified by genome-wide association analysis (GWAS) and linkage analysis, which provided a molecular basis for the development of molecular markers for developing waterlogging tolerant varieties. A recent study showed that *HvPRT6* RNAi improved the barley waterlogging tolerance by modulating *ERFVII*, which acts as a substrate for the n-terminal rule pathway [10].

RNA-Seq (RNA-sequencing), known as transcriptome sequencing, is a well-established technology for transcriptome analysis using next-generation sequencing technology, which has been successfully applied to several plants, including kiwifruit [11], tomato [12], maize [13], and rice [14], for mining genes related to flooding or waterlogging tolerance. In barley, several RNA-Seq studies related to waterlogging tolerance have been reported. Wang et al. [15] reported the key differentially expressed genes (DEGs) regulating waterlogging tolerance using the leaves and roots of two barley varieties with contrasting waterlogging tolerance using RNA-Seq. Luan et al. [16] compared the DEGs of two barley varieties at different periods under waterlogging stress, and showed that the DEGs were mainly enriched in the energy metabolism, hormone regulation, active oxygen scavenging, and cell wall modification enzymes, with ethanol dehydrogenase (ADH) playing an important role in coping with waterlogging. Luan et al. [17] identified 27 candidate genes associated with waterlogging, including 3 transcription factor (TF) genes (*HvDnaJ*, *HvMADS*, and *HvERF1*), by combining GWAS and RNA-Seq.

In this study, the DH line TamF169, with excellent waterlogging tolerance, and its sensitive parent Franklin were used for RNA-Seq analyses and physiological studies. We aimed to (1) screen differentially expressed genes in the root system of the two barley genotypes with contrasting tolerance in response to waterlogging, and (2) understand the mechanisms of the root system in response to waterlogging stress, which will provide a theoretical basis for the further excavation of waterlogging tolerance genes and the improvement of barley waterlogging tolerance.

## 2. Results

### 2.1. High-Throughput Sequencing of Root RNA Samples

The transcriptome sequencing and transcriptome profiling of the root samples from TamF169 and Franklin under control and waterlogging stress for 4 and 7 days were performed. After the quality control, a total of 148.23 Gb of clean data was obtained, with each sample yielding more than 19,050,380 clean reads. The GC content was between 52.02% and 53.48%, and the percentage of Q30 bases of each sample was greater than 93.64%, indicating that the high-throughput sequencing data were suitable for further transcriptome analysis (Table S1).

When the data were aligned to the barley reference genome (Hordeum_vulgare. MorexV3), the number of mapped reads was above 85.17%, and the number of uniquely mapped reads was above 82.98%, which indicated that the sequencing results had a high similarity and consistency with the reference genome (Table S2).

**Figure 1:**
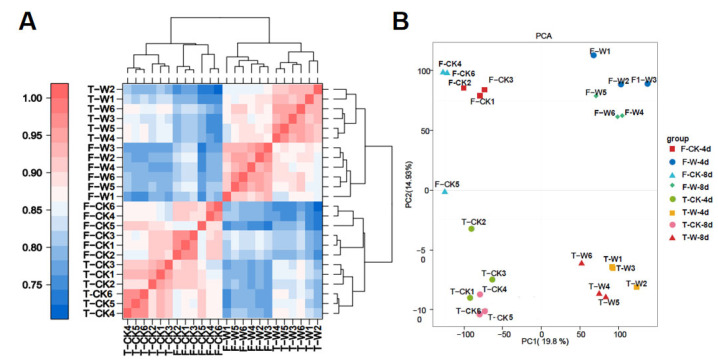
Correlation and principal component analysis of all of the samples. (**A**): correlation analysis of all of the samples; (**B**): principal component analysis of all of the samples.

Correlation analysis showed that the correlation coefficients’ r^2^ between biological replicates were all above 0.8 (Figure 1A). Principal component analysis (PCA) showed that the expression patterns within the same treatment for a single material were relatively clustered, whereas the PCA distances between the different treatments or varieties were significantly wider (Figure 1B).

### 2.2. Screening and Analysis of DEGs in Roots in Response to Waterlogging Stress

**Figure 2:**
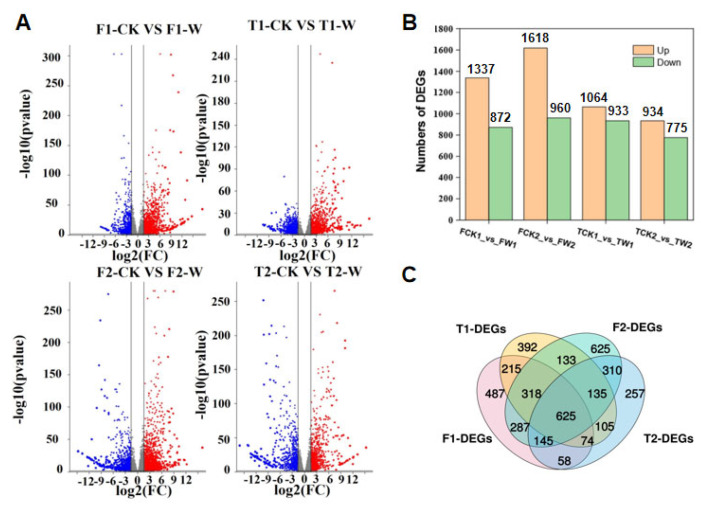
Differentially expressed genes (DEGs) in 4 groups (F1−CK vs. F1−W, F2−CK vs. F2−W, T1−CK vs. T1−W, and T2−CK vs. T2−W). (**A**): Volcano plot showing the DEGs in 4 groups. Red color represents up-regulated DEGs, blue color represents down-regulated DEGs, and gray color represents non-differentially expressed genes. (**B**): Histogram of DEGs in 4 groups. (**C**): Venn diagram of DEGs in 4 groups.

In order to identify the significant differentially expressed genes (DEGs) of the two genotypes under waterlogging stress, the expression genes of 24 samples under waterlogging for 4 and 8 days were analyzed with the criteria of FDR < 0.05 and Fold Change ≥ 3. The DEGs were identified through comparing the differential expression levels of genes in the following four groups: F1-CK vs. F1-W (Franklin under control and waterlogging for 4 days), F2-CK vs. F2-W (Franklin under control and waterlogging for 8 days), T1-CK vs. T1-W (TamF169 under control and waterlogging for 4 days), and T2-CK vs. T2-W (TamF169 under control and waterlogging for 8 days). In total, 2209 (1337 up-regulated and 872 down-regulated genes) and 2578 (1618 up-regulated and 960 down-regulated genes) DEGs were detected through comparing the gene expression levels in Franklin under control and waterlogging for 4 and 8 days, respectively. In total, 1997 (1064 up-regulated and 933 down-regulated genes) and 1709 (934 up-regulated and 775 down-regulated genes) DEGs were detected through comparing the gene expression levels in TamF169 under control and waterlogging for 4 and 8 days, respectively (Figure 2A,B). The comparison of the DEGs from these four groups identified 626 common differentially expressed genes, which may be genes that respond to waterlogging stress. In contrast to the common DEGs, 487 and 625 DEGs were specific only in Franklin, whereas 392 and 257 DEGs were specific only in TamF169 under waterlogging for 4 and 8 days, respectively (Figure 2C).

### 2.3. Functional Enrichment of GO and KEGG Analysis

**Figure 3:**
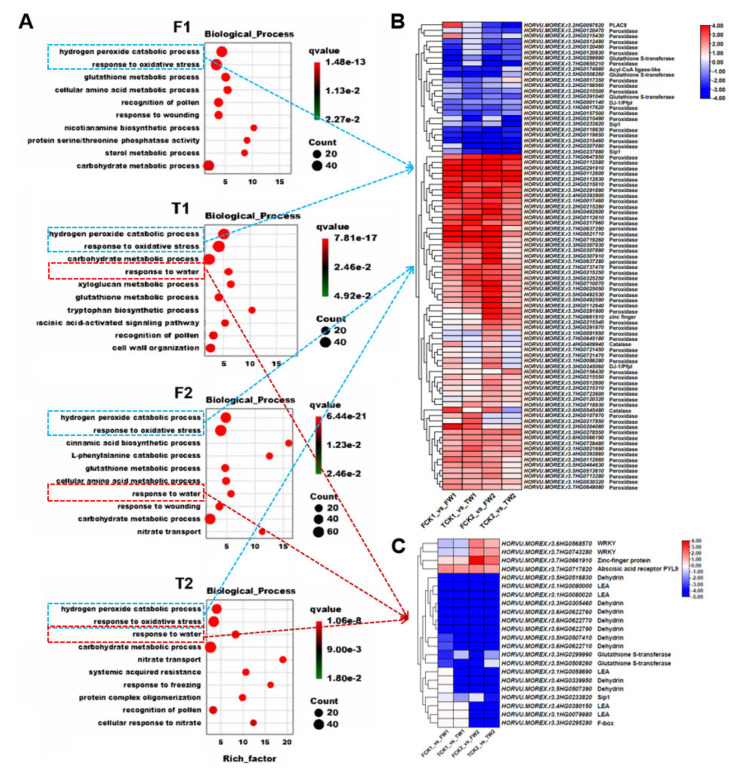
The GO enrichment of DEGs on the biological process. (**A**): The GO enrichment of DEGs on the biological process, (**B**): DEGs in the 2 terms of hydrogen peroxide catabolic process and response to oxidative stress. (**C**) DEGs in the term of response to water.

In order to understand the GO entries that were significantly enriched compared to the whole genomic background, the differential gene sets for each subgroup were analyzed for the enrichment of the biological processes, molecular functions, and cellular components, respectively, using ClusterProfiler employing hypergeometric tests. The term obtained from the enrichment results were visualized using a bubble chart (Figure S1). Cellular components were mainly enriched in the extracellular region, apoplast, anchored component of the plasma membrane, and cell wall. In terms of molecular functions, the genes were mainly enriched in heme binding, oxidase activity, iron ion binding, and the incorporation of one atom of oxygen. For the biological process classification, both the top two enrichment terms in the two genotypes are related to hydrogen peroxide catabolic process, which mainly includes the differentially expressed genes encoding peroxidase. Notably, after 4 days of waterlogging stress, more genes were specifically enriched on the waterlogging tolerance genotype TamF169, while, after 8 days of waterlogging stress, similar enriched genes were found in both genotypes (Figure 3). In the term of response to waterlogging, genes encoding LEA (late embryogenesis abundant protein) and dehydrin, such as HORVU.MOREX.r3.1HG0058690, HORVU.MOREX.r3.4HG0339950, and HORVU.MOREX.r3.5HG0507390, were significantly decreased only in the waterlogging-tolerant genotype TamF169.

**Figure 4:**
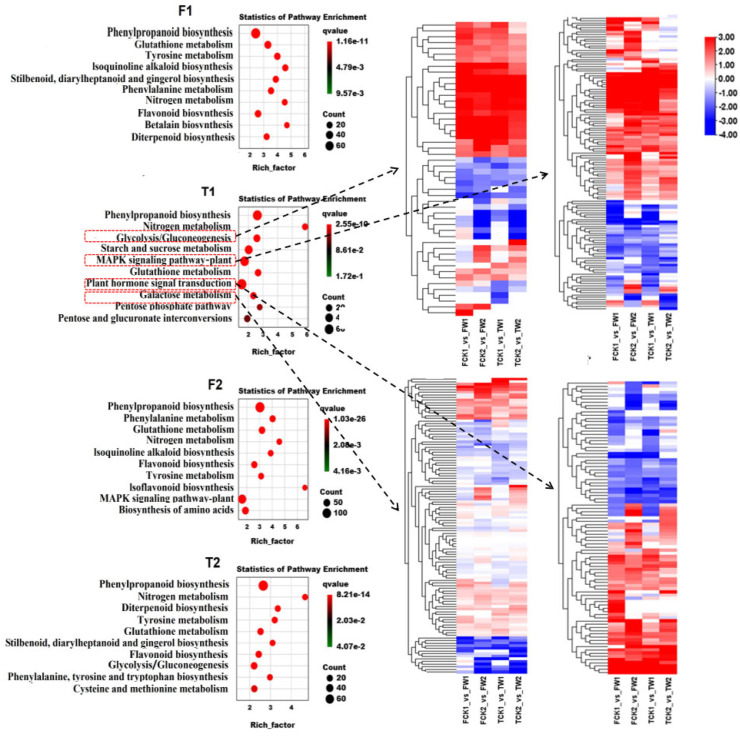
KEGG pathway enrichment results of the DEGs.

To further investigate the molecular mechanism of waterlogging tolerance in barley, the analysis of the KEGG pathway enrichment was performed. Glycolysis/gluconeogenesis, the MAPK signaling pathway, plant hormone signaling, and galactose metabolism pathways were among the top 10 pathways which were significantly enriched in the waterlogging-tolerant genotype TamF169 after waterlogging for 4 days, including 50, 130, 93, and 113 DEGs, respectively (Figure 4).

### 2.4. Candidate Gene Analysis for RCA−QTL Conferring Waterlogging Tolerance

**Figure 5:**
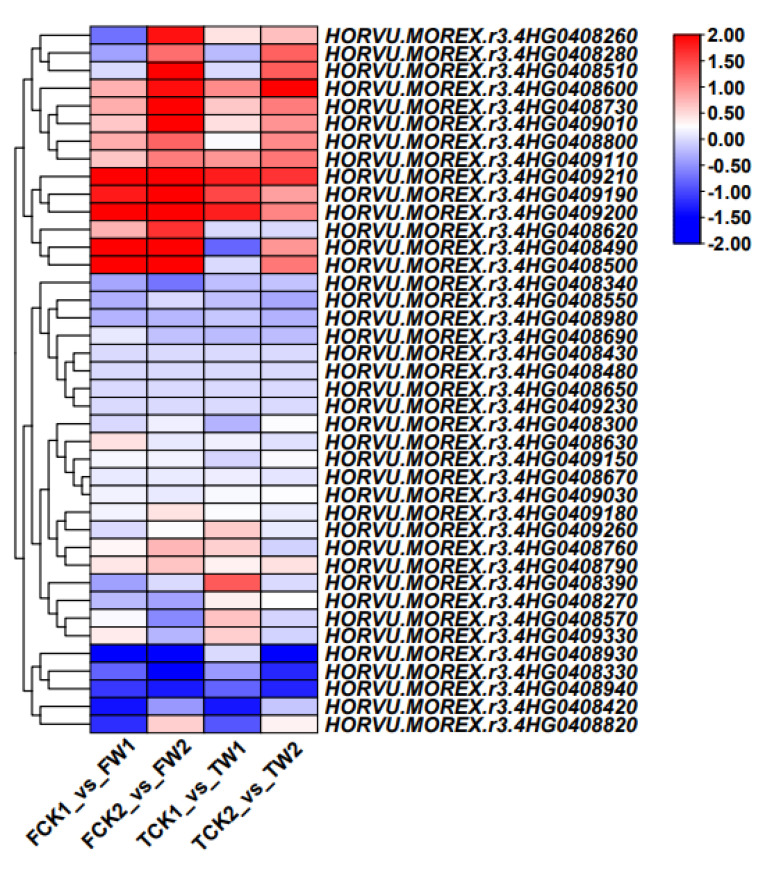
DEGs in the mapping region of *RCA*−*QTL*.

The root cortical aerenchyma RCA−QTL was localized between 4415244S4 (97.5 cM) and 6280297D4 (99.1 cM) [18], corresponding to the physical positions 581,599,730 bp and 585,620,814 in the barley genome (Hordeum vulgare. MorexV3). In this region, 40 genes were identified by RNA-Seq using the stringent filtering criterion of an FPKM value of 1.0 in this mapping region. Among the 40 genes, 13 genes showed different expression levels in Franklin or TamF169 after waterlogging stress (Figure 5 and Table S3). HORVU.MOREX.r3.4HG0409010, encoding a basic helix–loop–helix (bHLH) transcription factor, was highly induced in Franklin after 8 days of waterlogging. The KEGG pathway annotation of HORVU.MOREX.r3.4HG0409010 was the MAPK signaling pathway (ko04016) and plant hormone signal transduction (ko04075). HORVU.MOREX.r3.4HG0409010 was enrichened in the KEGG pathway of the MAPK signaling pathway and plant hormone signal transduction (Figure 5, Table S3). LEA (HORVU.MOREX.r3.4HG0408930) encoded a late embryogenesis abundant protein, which was significantly down-regulated in the expression in Franklin and TamF169 after 4 and 8 days of waterlogging, respectively (Figure 5 and Table S3). Therefore, *bHLH* and LEA were considered as the potential candidate genes for *RCA*−*QTL*.

### 2.5. qPCR Validation

**Figure 6:**
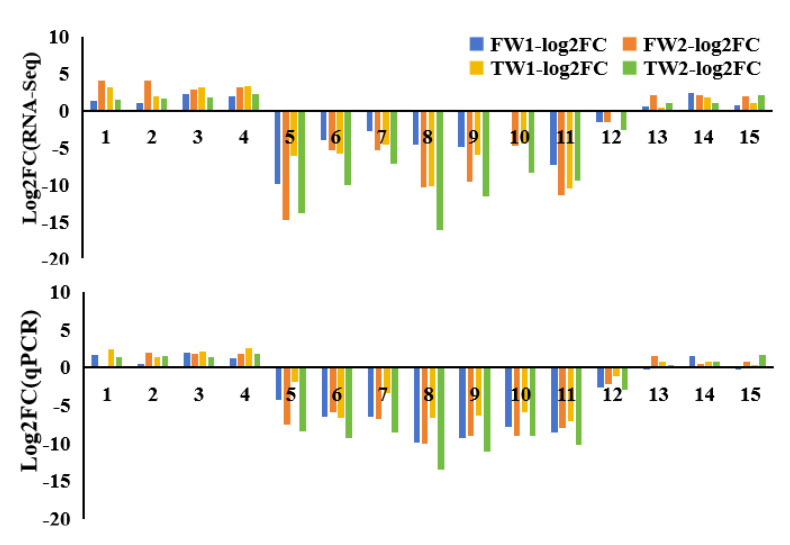
The qPCR validation of 15 differentially expressed genes in the RNA−Seq data.

To validate differentially expressed genes identified in the RNA sequencing data, 15 DEGs were selected for the qRT-PCR analysis. These DEGs were HORVU.MOREX.r3.7HG0710070 (1, peroxidase), HORVU.MOREX.r3.2HG0112640 (2, peroxidase), HORVU.MOREX.r3.2HG0215250 (3, peroxidase), HORVU.MOREX.r3.3HG0325250 (4, peroxidase), HORVU.MOREX.r3.3HG0305460 (5, dehydrin), HORVU.MOREX.r3.5HG0516830 (6, dehydrin), HORVU.MOREX.r3.6HG0622710 (7, dehydrin), HORVU.MOREX.r3.6HG0622760 (8, dehydrin), HORVU.MOREX.r3.6HG0622770 (9, dehydrin), HORVU.MOREX.r3HG0058690 (10, late embryogenesis abundant protein), HORVU.MOREX.r3HG0080000 (11, late embryogenesis abundant protein), HORVU.MOREX.r3.4HG0408330 (12, WAT1-related protein), HORVU.MOREX.r3.4HG0409010 (13, basic helix–loop–helix transcript factor), HORVU.MOREX.r3.4HG0409200 (14, glutathione S-transferase), and HORVU.MOREX.r3.4HG040860 (15, GDSL esterase/lipase). This qRT-PCR result was consistent with the transcriptome data (Figure 6).

## 3. Discussion

Waterlogging stress is one of the major factors limiting crop production. However, due to the low heritability of waterlogging tolerance in crops and the difficulty of phenotypic identification, the breeding of waterlogging-tolerance barley using traditional strategies is difficult [19,20]. Introgressing waterlogging-tolerance genes into commercial varieties is considered as the most economical way to reduce losses. The adoption of waterlogging-tolerant genotypes could mitigate the yield penalty caused by waterlogging for up to 18% under future climate conditions, suggesting that further research and development of such genotypes is a worthwhile investment [4]. TamF169, a double haploid (DH) line from a cross between Franklin and a wild barley genotype TAM407227, carries an *RCA*−*QTL* for waterlogging tolerance on 4H, which is responsible for aerenchyma formation under waterlogging stress, and shows significantly better waterlogging tolerance [18]. Under waterlogging stress, compared to Franklin, TamF169 showed a significantly higher yield, higher root cortical aerenchyma, and more adventitious roots [21]. The average yield increase in the three *RCA*−*QTL* introgressed tolerant lines (the genotypic background of backcross was three commercial varieties) was 1.8 t/ha under waterlogged conditions, while the addition of *RCA*−*QTL* showed no significant negative effects on plant growth, yield, and grain quality attributes under control conditions [21,22]. Therefore, the *RCA*−*QTL* is a promising breeding target for mitigating losses caused by waterlogging. In the mapping region of *RCA*−*QTL* (581,599,730 bp to 585,620,814 bp) [18], a bHLH transcription factor (*HORVU.MOREX.r3.4HG0409010*) enrichened in the KEGG pathway of the MAPK signaling pathway and plant hormone signal transduction, was highly induced in Franklin after waterlogging. In plants, bHLH factors are crucial for diverse signaling networks, plant development, and stress responses [23,24,25]. In grapes (*Vitis vinifera*), a bHLH factor, *VvbHLH036*, plays an important role in cold stress response by regulating threonine biosynthesis [26]. Drought-induced *OsbHLH130* can activate *OsWIH2*, improving drought resistance by participating in epidermal wax biosynthesis and reducing the water loss rate as well as ROS accumulation in rice [27]. *OsbHLH6*, which is involved in SA and JA signaling through nuclear cytoplasmal transport, endows rice with disease resistance [28]. The *bHLH* was identified as a core gene for underlying waterlogging stress responses by co-expressing modules related to flood resistance in quinoa seedlings [29]. The overexpression of *CabHLH18* in hot peppers could significantly enhance the overall waterlogging tolerance [30]. Therefore, the bHLH factor *HORVU.MOREX.r3.4HG0409010* was recognized as one of the potential candidate genes for *RCA−QTL*.

LEA proteins belong to a large group of proteins known as “hydrophilic proteins”, which are characterized by a glycine-rich, highly hydrophilic disordered amino acid sequence that can be classified into seven distinctive groups. Dehydrins belong to the group 2 LEA proteins, which play multifunctional roles in plant stress tolerance. LEA proteins are confirmed to play important roles in plant growth and development, and in mitigating the deleterious effects of various adversity conditions. Under desiccation or under extreme temperature conditions such as drought, cold, heat, and salinity, the expression of LEA has been shown to be significantly induced [31]. Dehydrins are now thought to be ubiquitous and expressed in plants after exposure to dehydration stress [32]. The LEA-3 group gene HVA1 was widely studied in barley, which played a very important role in maintaining the stability of the cell membrane under drought and salt stress, therefore protecting the cell against dehydration and desiccation. The heterologous overexpression of HVA1 in rice, maize, oat, mulberry, and creeping bentgrass, a common bean, differentially increased drought tolerance or salt tolerance in transgenic plants [33]. The function of LEA proteins with hydrophilic and osmoprotective effects has been widely reported, but the role of LEA in the presence of excess water has not been mentioned. In this study, the expression of LEA proteins, including dehydrin, was substantially down-regulated under waterlogging stress, exhibiting an expression pattern opposite to that under other stresses. Whether this is a stress response of the plant in the face of excess water or a protective effect on the plant needs to be further investigated. Therefore, *LEA* (HORVU.MOREX.r3.4HG0408930), located in the RCA−QTL mapping region and differentially expressed after waterlogging stress, was another candidate gene.

Under waterlogging stress, plant roots gradually shift from aerobic to anaerobic respiration to maintain intracellular energy levels. The transcripts of the genes related to the glycolytic and anaerobic fermentation pathway, such as alcohol dehydrogenase (ADH) and pyruvate decarboxylase (PDC), were significantly up-regulated in response to hypoxia, which provided the necessary energy for plant growth [34]. Studies on several plants, such as barley [15,16], rice [35], cucumber [36], wheat [37], and kiwifruit [38], revealed that the expression of ADHs under hypoxia stress were significantly induced. In this study, seven genes encoding ADH were highly induced in two genotypes. The importance of MAPK and hormone signaling pathways in the response to waterlogging stress has been reported in the transcriptome analysis of plant response to waterlogging stress [39,40,41]. Flooding/hypoxia activates *MPK3/6*, which then phosphorylate the *RAP2.3* (ERF-VII transcription factor). Compared to the wild type, *MPK3* and *MPK6* mutants were more sensitive to hypoxia and submergence stress, and the activation of *MPK3/6* upon hypoxia involved the phospholipase D-dependent release of phosphatidic acid [42,43]. Phosphatidic acid (PA) modulates submergence tolerance via both membrane integrity and *MPK3/6*-mediated hypoxia signaling in Arabidopsis [44]. The tolerance allele *SUB1A1* binds to the *MPK3* promoter to form the *MPK3*-*SUB1A1* module, which plays a crucial role in regulating submergence [43]. The gaseous phytohormone ethylene accumulates rapidly in plant tissues under waterlogged conditions, which is an important regulator of plant waterlogging tolerance [45]. Oxygen deficiency in the root system under waterlogging stress leads to the disruption of endogenous hormone levels in the plant levels, resulting in a reduction in the plant’s ability to absorb water and the nutrient capacity [46]. Furthermore, several plant hormones are intricately linked to ethylene, and, in particular, the crosstalk and interactions between ethylene and ABA, GA, and IAA are critical for plant flooding tolerance [47]. In this study, and consistent with these previous studies, glycolysis/gluconeogenesis, the MAPK signaling pathway, and plant hormone signaling were identified as the vital pathways involved in the response to waterlogging in the tolerant genotype TamF169.

## 4. Materials and Methods

### 4.1. Plant Materials and Waterlogging Treatment

TamF169, showing superior waterlogging tolerance, is a DH line from a DH population constructed by the cultivated barley Franklin and the wild barley TAM407227 [19]. In the DH population, a major-effect QTL (*RCAQTL*) for waterlogging tolerance and aerenchyma formation in wild barley was identified.

The seeds of TamF169 and Franklin were placed on wet filter paper and germinated in a constant temperature incubator (25 °C, protected from light) for 24 h. Well-geminated seeds were then transplanted to pots (7 cm × 7 cm × 12 cm) filled with potting mix (four seeds in each pot) in a greenhouse of Yangzhou University. The potting mix is a cultivation substrate consisting of peat, coir, vermiculite, and perlite mixed in certain proportions. When the plants grew to the three-leaf stage, the pots were placed in a water tank for the waterlogging treatment, with the water level being 2~3 cm above the surface. The controls were sown in a well-drained container. After 3 and 7 days of the waterlogging treatment, the roots of two genotypes under control and waterlogging stress were washed and quickly put into liquid nitrogen, and then stored in an ultra-low-temperature refrigerator at −80 °C.

### 4.2. RNA Extraction, Library Construction, and Sequencing

The total RNA of TamF169 and Franklin was extracted from three biological replications of waterlogging-stressed and control root samples, and purified using the RNeasy mini kit. The quantity and quality of the isolated RNA was checked using a NanoDrop 2000 spectrophotometer and denaturing agarose gel electrophoresis, respectively. After the samples were qualified, the library construction was carried out. The Qubit 3.0 Fluorescence Quantification Instrument, a new generation of nucleic acid and protein quantifiers for the precise quantification of DNA and RNA, was then used for the preliminary quantification. Subsequently, the insertion fragment of the library was detected using the Qsep400 High-Throughput Analytical System. Qsep400 is a high-throughput nucleic acid protein analysis system that automatically calculates the fragment size for clearer and more accurate results. When the insertion fragment met the expectation, the qPCR method was used to accurately quantify the effective concentration of the library (the effective concentration of the library was >2 nM) to ensure the quality of the library. After the library passed the quality control, PE150 mode sequencing was performed using the Illumina NovaSeq6000 sequencing platform.

### 4.3. Quality Control of Sequencing Data

Raw data were generated by sequencing cDNA libraries using a high-throughput sequencing platform. To ensure a sufficiently high quality for accurate subsequent analyses, we performed strict quality control of the data by performing the following filtering methods: (1) removal of reads containing junctions; (2) removal of low-quality reads (including reads with a proportion of N greater than 10% and reads with more than 50% of the entire read in bases with a quality value of Q ≤ 10). The high-quality data obtained after a series of quality controls, as described above, were used for the subsequent analyses.

### 4.4. Analysis of Gene Expression

The resulting high-quality clean reads were compared to the reference genome Hordeum vulgare. MorexV3 (Index of/pub/plants/release-60/fasta/hordeum_vulgare/dna) was used for sequence comparison and subsequent analyses. We used HISAT2 software to compare the clean reads with the reference genome to obtain information on the localization of the reads on the reference genome [48]. The reads were then assembled using StringTie to reconstruct the transcriptome for the subsequent analysis [49]. In order to accurately reflect the expression level of the transcript, we used StringTie to normalize the number of fragments by the maximum flow algorithm using FPKM (Fragments Per Kilobase of transcript per Million fragments mapped) as a measure of the transcript or gene expression level. The stringent filtering criterion of the FPKM value of 1.0 was used to obtain the expressed transcripts.

### 4.5. Identification of DEGs

The DEGs were screened based on the count value of the genes in each sample using differential analysis software, DESeq [50]. Fold Change ≥ 3 and FDR < 0.05 were used as the screening criteria during the DEG detection. Fold Change indicates the ratio of expression between two samples (groups). The False Discovery Rate (FDR) was obtained by correcting the *p*-value (*p*-value) for the significance of difference. To facilitate comparison, the the multiplicity of differences was taken as a logarithmic value, expressed as log2FC. Gene Ontology (GO) and the KEGG enrichment analysis of the DEGs was performed by the Cluster Profiler R package in the R program version 4.4.1 [51], with corrected *p*-values of less than 0.05.

### 4.6. Quantitative Reverse Transcription Polymerase Chain Reaction (qPCR)

For the qPCR analysis, 2 μg of DNaseI-digested total RNA was used for the cDNA synthesis using HiScript^®^ III RT SuperMix (cat. no. R323; Vazyme Biotech Co., Ltd., Nanjing, China). The qPCR reactions were performed in 10 μL total reaction volumes using the ChamQ Universal SYBR^®^ qPCR Master Mix (cat. no. Q711; Vazyme Biotech Co., Ltd, China). ACTIN and GAPDH was used as the reference gene. The procedures for the qPCR were conducted as follows: 3 min at 95 °C; (10 s at 95 °C; 30 min at 60 °C) for 40 cycles. The data were analyzed with the 2^−ΔΔCt^ method to determine the relative gene expression level [52]. The primers used for the qPCR analysis are shown in Table S4.

## 5. Conclusions

Waterlogging stress poses a serious threat to barley production. It is critical to understand the mechanisms of barley waterlogging tolerance. In this study, high-throughput sequencing and thoroughly examined transcriptome changes after waterlogging for 0, 4, and 8 days in barley roots were performed. The KEGG analysis indicated significant enrichments in glycolysis/gluconeogenesis, the MAPK signaling pathway, plant hormone signaling, and galactose metabolism pathways in the waterlogging-tolerant DH line TamF169. A total of 13 genes in the mapping region of *RCA−QTL* showed different expression levels in Franklin or TamF169. Among them, the *bHLH* factor (HORVU.MOREX.r3.4HG0409010) and *LEA* (HORVU.MOREX.r3.4HG0408930) were considered as the potential candidate genes for *RCA−QTL*. This study offers valuable information on the mechanism of tolerance to waterlogging stress in the DH line TamF169 and provides the candidate genes for *RCA−QTL*.

## Data Availability

The data presented in this study are available upon request from the corresponding authors.

## Supplementary Materials

The following supporting information can be downloaded at: https://www.mdpi.com/article/10.3390/plants14010028/s1, Table S1: Statistical table of sequencing data; Table S2: Statistics of the sequence comparison results between the sample sequencing data and barley reference genomes; Table S3: DEGs in the mapping region of RCA−QTL; Table S4: Primers used for the qPCR analysis.
